# Supplementary material for: Combination of Local Ablative Techniques with Radiotherapy for Primary and Recurrent Lung Cancer: A Systematic Review
Source: Cancers (Basel). 2023 Dec 16;15(24):5869. doi: 10.3390/cancers15245869 (PMC10741973; doi:10.3390/cancers15245869)
Supplement: Supplementary file 1 [file cancers-15-05869-s001.zip › cancers-2663284-supplementary.pdf]

Study	Was hypothesis stated?			Was the study prospective?			Is multicentric?			Consecutive patients			Descriibed characteristics of patients			Clearly stated eligibility criteria			Described intervention			Reported losses to follow-up and adverse events			Conclusions of the study supported by the results			Final Score
	Yes	Partial	No	Yes	Unclear	No	Yes	Unclear	No	Yes	Unclear	No	Yes	Partial	No	Yes	Partial	No	Yes	Partial	No	Yes	Unclear	No	Yes	Unclear	No	
Steber (2021)	2	_	_	2	_	_	_	_	0	2	_	_	2	_	_	_	1	_	2	_	_	2	_	_	2	_	_	15
Grieco (2006)	2	_	_	_	_	0	_	_	0	2	_	_	2	_	_	_	1	_	2	_	_	2	_	_	2	_	_	13
Dupuy (2006)	2	_	_	_	_	0	_	_	0	2	_	_	2	_	_	_	1	_	2	_	_	2	_	_	2	_	_	13
Cheng (2016)	2	_	_	_	_	0	_	_	0	2	_	_	2	_	_	_	1	_	2	_	_	2	_	_	2	_	_	13
Leung (2010)	2	_	_	_	_	0	_	_	0	2	_	_	2	_	_	_	1	_	2	_	_	2	_	_	2	_	_	13
Brooks (2018)	2	_	_	_	_	0	_	_	0	2	_	_	_	1	_	_	1	_	2	_	_	_	1	_	_	1	_	10

Supplementary Table 1: *Quality appraisal score by Institute of Health Economics (IHE) for the selected studies*
